# Effect of dexmedetomidine combined with the modified Trendelenburg position on intraocular pressure in patients undergoing robot-assisted laparoscopic surgery: A randomized controlled trial

**DOI:** 10.1371/journal.pone.0345975

**Published:** 2026-03-27

**Authors:** Shuyang Yan, Jindong Liu, Beibei Xiao, Chunyan Guo, Binghan Wang, Runhan Shi, Qian Deng

**Affiliations:** 1 Department of Anesthesiology, The Affiliated Hospital of Nantong University, Nantong, Jiangsu, China; 2 Department of Anesthesiology, The Affiliated Hospital of Xuzhou Medical University, Xuzhou, Jiangsu, China; Alexandria University Faculty of Medicine, EGYPT

## Abstract

**Objective:**

This study aimed to evaluate the effect of combining dexmedetomidine infusion with a modified Trendelenburg position on intraocular pressure (IOP) in patients undergoing robot-assisted laparoscopic surgery (RALS).

**Methods:**

In a single-center, prospective, 2 × 2 factorial randomized controlled trial, 160 patients scheduled for RALS were allocated to one of four groups: control (normal saline + traditional position), modified position alone, dexmedetomidine alone, or the combination of both. IOP was measured at baseline, during key surgical phases, and after extubation. The primary outcome was IOP during surgery. Secondary outcomes included the incidence of cardiovascular adverse events and the requirement for vasoactive drugs.

**Results:**

A total of 153 patients completed the study protocol. Both dexmedetomidine and the modified position significantly reduced IOP during surgery (main effect of drug: F = 35.2, *P* < 0.001; main effect of position: F = 28.7, *P* < 0.001). Critically, a statistically significant interaction was observed between the two interventions (F = 4.8, *P* = 0.030), indicating a synergistic effect. The combination group demonstrated the greatest reduction in IOP from time points T2 to T4, ranging from 3.6 to 4.8 mmHg lower than the control group. There were no significant differences in the overall incidence of adverse events among the groups.

**Conclusions:**

In conclusion, for patients undergoing prolonged robotic laparoscopic surgery (with a mean operative time of 217 minutes), the combined use of dexmedetomidine infusion and a modified Trendelenburg position provides an effective and synergistic strategy for intraocular pressure control. This approach offers a practical means to enhance intraoperative ocular safety during such lengthy procedures.

**Trial registration:**

Chinese Clinical Trial Registry ChiCTR2300072961

## Introduction

Robotic-assisted laparoscopic surgery (RALS) enhances surgical precision and recovery but often requires prolonged steep Trendelenburg position (25–45° head-down tilt) under CO₂ pneumoperitoneum ^[^1–3^]^. This combination significantly elevates intraocular pressure (IOP), thereby posing a risk for perioperative glaucomatous injury and irreversible visual field loss ^[^4–7^]^.

Dexmedetomidine, a selective α₂-adrenoceptor agonist, reduces aqueous humor production and has shown promise in mitigating IOP rise ^[^8–11^]^. Concurrently, a modified Trendelenburg position, achieved by elevating the head and shoulders, may improve venous drainage and lower IOP without compromising surgical access ^[^12^]^.

As single interventions often provide suboptimal IOP control, this study aimed to investigate the combined effect of dexmedetomidine infusion and the modified Trendelenburg position on IOP in patients undergoing robot-assisted surgery. We hypothesized that their complementary mechanisms would yield a synergistic effect, offering an optimized strategy for anesthesia management and enhanced intraoperative ocular safety.

## Methods

### Ethical approval

This single-center, prospective, 2 × 2 factorial randomized controlled trial was approved by the Ethics Committee of the Affiliated Hospital of Xuzhou Medical University (XYFY2023-KL152–01) and registered at the China Clinical Trial Registry (ChiCTR2300072961). The study was conducted from June to November 2023. All eligible patients were fully informed of the study details and provided written informed consent prior to participation.

### Study subjects

**Fig 1:**
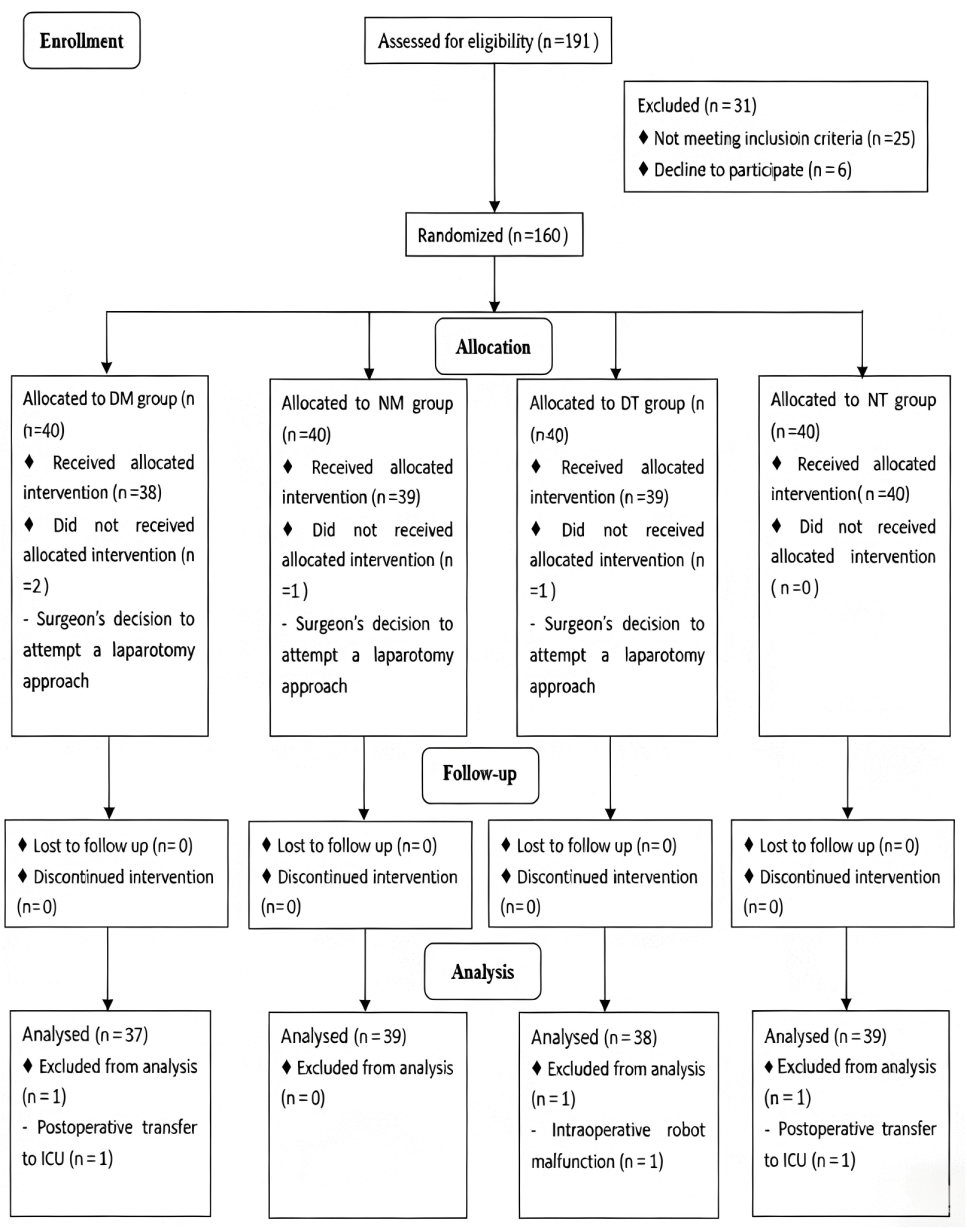
Flowchart of the participants included in the analysis.

Eligible participants were adults (≥18 years) with a BMI of 18–30 kg/m², an ASA physical status of I–II, and scheduled for robot-assisted laparoscopic radical resection of prostate, colorectal, or endometrial cancer. Exclusion criteria included: ophthalmic conditions (glaucoma, diabetic retinopathy, cataracts, or retinal detachment); baseline intraocular pressure (IOP)>30 mmHg; history of intraocular surgery; high myopia (spherical equivalent > –6.00 D); allergy to any study medication; cardiovascular disorders (hypotension, bradycardia, ventricular conduction abnormalities, or congestive heart failure); neurogenic disorders; or history of migraine (**Fig 1**).

### Randomization

Block randomization was performed with a block size of 8. An independent biostatistician generated the allocation sequence using SPSS software (version 27.0). Participants were assigned to blocks sequentially according to their enrollment order. Within each block, patients were randomly allocated in a 1:1:1:1 ratio to one of the four study groups. Allocation was concealed using sequentially numbered, opaque, sealed envelopes.

### Intervention

A total of 160 patients were randomly assigned to one of four groups: control (normal saline + traditional position, NT), modified Trendelenburg position alone (NM), dexmedetomidine alone (DT), or dexmedetomidine plus modified Trendelenburg position (DM). All study medications were prepared by an independent investigator not involved in IOP measurement or anesthetic management. Patients, surgeons, research staff, and managing anesthesiologists were blinded to group assignment.

Standard monitoring included invasive blood pressure (IBP), electrocardiogram (ECG), pulse oximetry (SpO₂), end-tidal carbon dioxide (PETCO₂) and bispectral index (BIS). Anesthesia was induced with sufentanil (0.5 µg/kg), etomidate (0.3 mg/kg), and rocuronium bromide (0.6 mg/kg). Maintenance was achieved with continuous infusions of propofol (0.8 mg/kg/h), remifentanil (0.05 µg/kg/min), and rocuronium bromide (0.6 mg/kg/h). Mechanical ventilation was set to a tidal volume of 6–8 ml/kg, respiratory rate of 12–16 breaths/min, I: E ratio of 1:1.5, FiO₂ of 0.4, and fresh gas flow of 2 L/min, adjusted to maintain ETCO₂ between 35–45 mmHg. Muscle relaxants were discontinued 30–40 minutes before the end of surgery, and tropisetron (2 mg) was administered for prophylaxis against postoperative nausea and vomiting (PONV).

Two anesthesiologists were involved: one (unblinded) prepared the study drugs, and the other (blinded), who was not involved in anesthetic management, collected all data. IOP was measured by a trained anesthesiologist using a rebound tonometer (Icare Finland Osakeyhtio, Finland). Three measurements were taken from the right and left eyes, and the average of six measurements was taken. Measurements were obtained at baseline (T0), 2 minutes after anesthesia induction (T1), and at 0.5, 1.5, and 2.5 hours after initiation of CO₂ pneumoperitoneum (T2, T3, T4), and 2 minutes after extubation (T5).

In the DM and DT groups, a dexmedetomidine infusion (0.4 µg/kg/h) was started immediately after endotracheal intubation and continued until the end of surgery. In the NM and NT groups, an equivalent volume of normal saline was infused as a placebo at the same rate. Patients in the DT and NT groups were placed in the traditional steep Trendelenburg position after establishing pneumoperitoneum, which was maintained throughout surgery. In the DM and NM groups, a modified Trendelenburg position was used, involving elevation of the head and shoulders toward the horizontal plane while maintaining the Trendelenburg tilt, from pneumoperitoneum until the end of the procedure.

Vasoactive drugs were administered to maintain blood pressure within ±20% of baseline and heart rate between 60–100 bpm. Phenylephrine was given if systolic blood pressure (SBP) fell below 90 mmHg or decreased by >20% from baseline; ephedrine was administered if SBP met the same criteria and heart rate was < 60 bpm. If SBP exceeded 140 mmHg or increased by >20% from baseline, anesthetic depth was increased or urapidil was given. Atropine was administered for heart rates below 50 bpm.

### Outcomes

The primary outcome was intraocular pressure (IOP). Secondary outcomes included the incidence of cardiovascular adverse events and the rate of vasoactive drug use. Complications potentially related to intraocular hypertension (e.g., eye pain, nausea, vomiting, dizziness, headache) within the first 24 hours postoperatively were recorded. All outcome assessments were performed by an anesthesiologist blinded to group allocation.

### Sample size calculation

The sample size was calculated based on data from our pilot study, which enrolled 60 patients (15 per group). The calculation was primarily driven by the observed mean ± standard deviation of intraocular pressure (IOP) at the T2, T3, and T4 time points. For the four groups (DM, NM, DT, NT), the mean IOP (mmHg) across time points was: 18.7, 20.2, 19.4, 22.9 at T2; 21.1, 21.8, 22.2, 25.3 at T3; and 22.8, 22.9, 23.3, 25.5 at T4, respectively. To detect the observed difference with a two-sided significance level (α) of 0.05 and a statistical power (1-β) of 0.90, a sample size calculation for comparing two independent means indicated that 35 patients were required per group. Given the 2 × 2 factorial design comprising four groups (DM, DT, NM, NT), the total estimated sample size was 140 patients. Accounting for a potential dropout rate of approximately 10%, a final sample of 160 patients (40 per group) was planned for enrollment.

### Statistical analysis

Statistical analyzes were performed using SPSS Statistics (version 27.0). Normally distributed measurement data are presented as mean ± standard deviation, skewed distribution data as median and interquartile range. A two-way factorial analysis of variance (ANOVA) was used to analyze the main effects of the two interventions (dexmedetomidine and position) and their interaction. For multi-group comparisons, the Kruskal-Wallis H test was employed, with Bonferroni correction for post-hoc pairwise comparisons. Categorical data were analyzed using the χ2 test or Fisher’s exact test. *P*<0.05 was considered statistically significant.

## Results

### Participant flow and baseline characteristics

From June 2023 to December 2023, 160 of 191 assessed individuals met eligibility criteria. Ultimately, 153 patients completed the study and were included in analysis (**Fig 1 Flowchart of the participants included in the analysis)**. Demographic and perioperative characteristics were comparable across all four study groups (**Table 1**).

**Table 1:** Baseline characteristics of the participants.

	DM group (*n* = 37)	NM group (*n* = 39)	DT group (*n* = 38)	NT group (*n* = 39)	*P* value
Age (years)	66 ± 8	67 ± 9	64 ± 7	66 ± 8	0.365
Gender, n(%)					0.207
Male	25(68%)	24(62%)	29(76%)	21(54%)	
Female	12(32%)	15(38%)	9(24%)	18(46%)	
BMI (kg/m^2^)	22.8 ± 2.1	23.2 ± 2.0	23.1 ± 1.7	23.1 ± 2.2	0.805
ASA classification, n (%)					0.807
Ⅰ	10(27%)	8(21%)	12(32%)	9(23%)	
Ⅱ	27(73%)	31(79%)	26(68%)	30(77%)	
Type of operation, n(%)					0.068
Radical resection of prostate cancer	16(%)	25%(%)	18(%)	15(%)	
Radical resection of rectal cancer	17(%)	7(%)	15(%)	13(%)	
Radical operation of endometrial carcinoma	4(%)	7(%)	5(%)	11(%)	
Pneumoperitoneum pressure [mmHg, *M* (Q_1_*, Q*_*3)*_]	13 (13，14)	13 (13，14)	13(13，14)	14 (13，14)	0.708
Tilted angle [º, *M* (*Q*_*1*_*, Q*_*3*_)}	30 (30，35)	30 (30，35)	35 (30，35)	30 (30，35)	0.638
Time for surgery (AM/PM)	22/15	21/18	20/18	19/20	0.825
Duration, pneumoperitoneum (min)	162 ± 24	170 ± 25	160 ± 28	168 ± 22	0.277
Duration, Trendelenburg(min)	143 ± 22	152 ± 25	145 ± 27	155 ± 23	0.143
Duration, surgery (min)	217 ± 27	226 ± 24	223 ± 25	228 ± 22	0.271
Fluid intake (ml)	1559 ± 165	1575 ± 200	1498 ± 213	1605 ± 224	0.128
Blood loss (ml)	243 ± 108	263 ± 110	268 ± 131	273 ± 102	0.674
Urine output (ml)	254 ± 77	294 ± 93	277 ± 86	290 ± 86	0.177

BMI: body mass index; ASA: American Society of Anesthesiologists.

### Primary outcome: intraocular pressure

Both dexmedetomidine infusion and the modified Trendelenburg position significantly reduced IOP (main effects: F=35.2, *P*<0.001 and F=28.7, *P*<0.001, respectively). Critically, a statistically significant interaction between the two interventions was observed at time points T2, T3, and T4 (all *P*<0.05), but not at baseline (T0), induction (T1), or postoperatively (T5) (**Table 2**).

**Table 2:** The interactive effect of intraoperative pressure.

	T0	T1	T2	T3	T4	T5
NT group (n = 39)	16.7 ± 2.0	11.6 ± 1.8	23.1 ± 2.2	25.0 ± 1.8	25.2 ± 1.8	18.4 ± 1.9
DT group (n = 38)	17.1 ± 2.0	11.6 ± 2.0	20.4 ± 1.7	22.0 ± 1.9	23.1 ± 2.0	17.3 ± 2.0
NM group (n = 39)	16.9 ± 2.3	11.4 ± 1.8	20.5 ± 1.8	21.5 ± 2.0	23.0 ± 1.8	17.4 ± 2.1
DM group (n = 37)	16.7 ± 2.2	12.0 ± 1.8	16.5 ± 2.0	17.2 ± 2.0	19.4 ± 1.8	17.1 ± 2.0
*P* value, the main effect of drugs	0.709	0.293	＜0.001	＜0.001	0.001	0.030
Difference (95% *CI*), the main effect of drugs	0.10 [−0.59, 0.80]	0.31 [−0.28, 0.91]	3.4 [2.6, 4.2]	3.6 [2.7, 4.5]	2.8 [2.1, 3.6]	0.70 [0.06, 1.34]
*P* value, the main effect of various positions	0.727	0.713	＜0.001	＜0.001	＜0.001	0.043
Difference (95% *CI*), the main effect of various positions	0.35 [−0.35, 1.04]	0.10 [−0.50, 0.70]	3.2 [2.4, 4.1]	4.1 [3.3, 5.0]	2.9 [2.2, 3.7]	0.66 [0.02, 1.30]
*P* value, the interaction effect	0.853	0.423	0.043	0.046	0.011	0.224

Values are expressed as mean ± standard deviation. A two-factor analysis of variance (ANOVA) was employed for statistical analysis. If the interaction effect was significant, post-hoc tests were conducted to examine simple effects among the four groups. Otherwise, main effects between different interventions within each group were evaluated.

**Fig 2:**
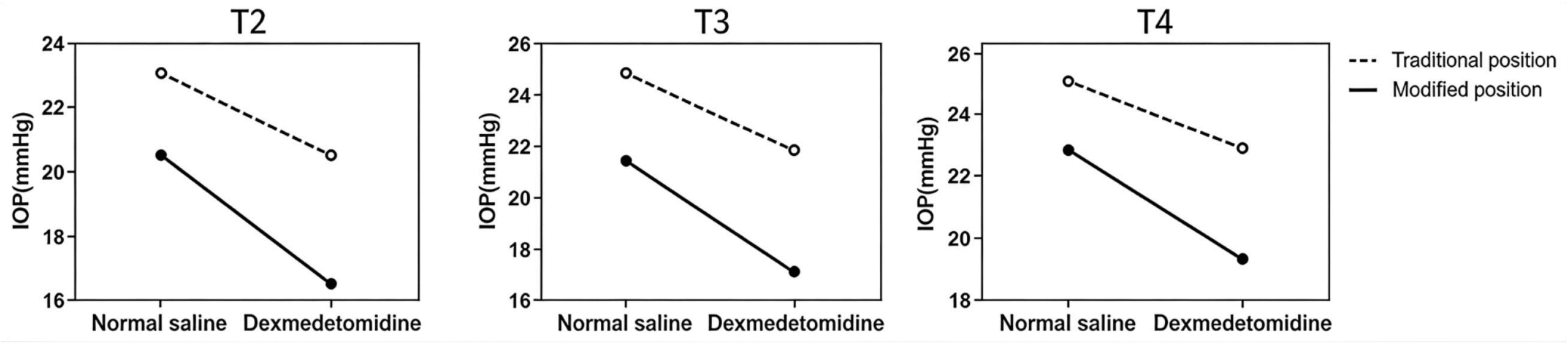
Interaction effect plot at T2,T3,T4. The parallel line indicates that there is no interaction between the two independent variables; Non-parallel and even crossing lines indicate the existence of interaction. If the effect of one independent variable is enhanced at a certain level of the other independent variable, it indicates a positive interaction exists.

The significant interaction (detailed in **Table 3** and visualized in **Fig 2 Interaction effect plot at T2,T3,T4**) indicates that the IOP-lowering effect of dexmedetomidine depended on patient positioning. Specifically, the IOP-lowering efficacy of dexmedetomidine was significantly greater when combined with the modified Trendelenburg position than with the traditional position. For instance, at T2, dexmedetomidine was associated with an additional reduction of 4.0 mmHg [95% CI: 3.2, 4.9] in the modified position, compared to 2.8 mmHg [95% CI: 1.9, 3.6] in the traditional position. This differential effect confirms a synergistic interaction between the two interventions.

**Table 3:** Simple effect at T2, T3, T4.

		Normal Saline	Dexmedetomidine	*P* value	*Difference (95*% CI)
T_2_	Modified position	20.5 ± 1.8	16.5 ± 2.0	＜0.001	4.0 [3.2, 4.9]
	Traditional position	23.1 ± 2.2	20.4 ± 1.7	＜0.001	2.8 [1.9, 3.6]
	***P*** value	＜0.001	＜0.001		
	*Difference (95*% ***CI***)	2.6 [1.8, 3.5]	3.9 [3.0, 4.8]		
T_3_	Modified position	21.5 ± 2.0	17.2 ± 2.0	＜0.001	4.3 [3.4, 5.2]
	Traditional position	25.0 ± 1.8	22.0 ± 1.9	＜0.001	3.0 [2.2, 3.9]
	***P*** value	＜0.001	＜0.001		
	*Difference (95*% ***CI***)	3.5 [2.7, 4.4]	4.8 [3.9, 5.7]		
T_4_	Modified position	23.0 ± 1.8	19.4 ± 1.8	＜0.001	3.6 [2.8, 4.5]
	Traditional position	25.2 ± 1.8	23.1 ± 2.0	＜0.001	2.1 [1.3, 2.9]
	***P*** value	＜0.001	＜0.001		
	*Difference (95*% ***CI***)	2.2 [1.3, 3.0]	3.7 [2.9, 4.6]		

### Safety outcomes

The safety profile is summarized in **Table 4**. No serious adverse events related to the study interventions were reported. The overall incidence of intraoperative cardiovascular events (hypotension, hypertension, or bradycardia) did not differ significantly among the four groups (*P*>0.05). Bradycardia was most frequent in the dexmedetomidine plus modified Trendelenburg group(19%), though the inter-group difference was not statistically significant (*P*=0.070). All events considered potentially related to the study drug or anesthetic management were transient and were conventionally managed with standard vasoactive agents. The requirement for vasoactive drug support also showed no significant difference across groups (*P*=0.636). Postoperative symptoms within 24 hours were comparable across groups, and notably, no cases of perioperative visual loss (POVL) occurred.

**Table 4:** Incidence of Adverse Events by Study Group.

	DM group (*n* = 37)	NM group (*n* = 39)	DT group (*n* = 38)	NT group (*n* = 39)	*P* value
Cardiovascular Event, n(%)	13 (35%)	11 (28%)	13 (33%)	11 (28%)	0.934
Hypotension, n(%)	4(11%)	6(15%)	5(13%)	5(13%)	0.987
Hypertension, n(%)	2(5%)	3(8%)	3(8%)	5(13%)	0.544
Bradycardia, n(%)	7(19%)	2(5%)	5(13%)	1(3%)	0.070
Vasoactive drugs, n(%)	5(14%)	8(20%)	6(16%)	9(23%)	0.746
Ocular pain, n(%)	1(3%)	1(3%)	1(3%)	2(5%)	1.000
Nausea and vomiting, n(%)	6(16%)	7(18%)	5(13%)	8(21%)	0.875
Dizziness and headache, n(%)	3(8%)	4(10%)	5(13%)	3(8%)	0.877

## Discussion

This study confirmed that in patients undergoing robot-assisted laparoscopic surgery with pneumoperitoneum in the Trendelenburg position, both dexmedetomidine administration and use of a modified Trendelenburg position independently reduced intraocular pressure (IOP). The pivotal finding is the significant synergistic interaction between these interventions, which was superior to that of either intervention alone.

We propose that the observed synergy arises because the two interventions act via complementary and mutually enhancing physiological pathways. The modified Trendelenburg position establishes a foundational advantage by improving venous drainage and reducing episcleral venous pressure ^[^11^]^, thus decreasing aqueous outflow resistance. Within this optimized setting, dexmedetomidine exerts a dual action—directly inhibiting aqueous production via peripheral α₂-receptors and, through central sympatholysis [12], reducing systemic drivers of inflow and vascular pressure—which amplifies the postural benefit on venous return. Consequently, the mechanically favorable state potentiates the drug’s pharmacologic effect, resulting in superior IOP control.

Methodologically, while Goldmann applanation tonometry is the clinical gold standard for IOP measurement ^[^13,14^]^, rebound tonometry was chosen for its practicality in the intraoperative setting. It is simpler, faster, does not require topical anesthesia, and is validated for use in non-sitting positions ^[^15–17^]^, with strong correlation to Goldmann measurements demonstrated in previous studies ^[^18–20^]^. Furthermore, our study enrolled a clinically diverse cohort undergoing different oncologic procedures. Although this heterogeneity might increase physiological variability, the observed IOP standard deviations were comparable to other studies in prolonged robotic surgery. All patients shared the primary risk factor of prolonged pneumoperitoneum and steep Trendelenburg positioning. The demonstration of significant IOP reduction and a clear synergistic effect despite this diversity underscores the robustness of our findings and enhances their external validity.

Although this study did not detect statistically significant differences in immediate postoperative IOP-related complications, this finding should be interpreted within two important limitations. First, the observation period was confined to 24 hours post-surgery, which may be insufficient to capture later-onset sequelae. Second, the intraoperative rise in IOP is typically transient. However, the absence of short-term clinical sequelae does not negate the established pathophysiological risk. Prolonged IOP elevation remains a well-documented risk factor for serious complications such as perioperative visual loss ^[^21,22^]^. Therefore, proactive intraoperative management remains a clinically prudent objective.

This has profound clinical relevance. For prolonged RALS, achieving adequate IOP control with a single agent often necessitates higher doses, increasing the risk of hemodynamic side effects. Our findings advocate for a combined, low-dose strategy. By harnessing synergy, clinicians can achieve effective ocular protection using a lower dose of dexmedetomidine—minimizing its adverse effect profile—while simultaneously employing a simple, cost-free postural adjustment. This integrated approach represents a practical and safe optimization of anesthesia management for high-risk surgery.

Regarding safety, while the overall incidence of cardiovascular events did not differ significantly, a higher numerical rate of bradycardia was observed in the dexmedetomidine group (19%). Although this did not reach statistical significance in our study, which was not powered for adverse event comparisons, this trend aligns with the known pharmacological profile of the drug. Therefore, heart rate monitoring remains advisable when employing this dexmedetomidine-based strategy.

## Limitations

This study has limitations. First, although rebound tonometry is well-validated against the Goldmann applanation tonometry gold standard ^[^18,19^]^, operational challenges in the intraoperative setting—such as ensuring consistent perpendicularity to the cornea—may introduce slight measurement deviations. Second, excluding glaucoma patients limits direct extrapolation of safety to that high-risk population; future studies should include them. Third, while we demonstrated physiological efficacy, the study was not powered to detect differences in rare clinical outcomes like POVL. Finally, the impact of the modified position on surgical ergonomics was not assessed, an important consideration for implementation.

## Conclusion

The synergistic combination of dexmedetomidine and a modified Trendelenburg position provides superior intraocular pressure reduction compared to either intervention alone in patients undergoing robot-assisted laparoscopic surgery.
